# Catheter ablation versus antiarrhythmic drug therapy for sustained ventricular tachycardia in patients with hypertrophic cardiomyopathy

**DOI:** 10.1186/s12872-024-03924-w

**Published:** 2024-05-16

**Authors:** Yan Dong, Xudong Song, Dan Bo, Hongtao Wang, Bo Yang, Nishant Yadav, Qiushi Chen, Ruochen Xu, Hongwu Chen, Weizhu Ju, Kejiang Cao, Minglong Chen, Fengxiang Zhang

**Affiliations:** 1 Section of Pacing and Electrophysiology, Division of Cardiology, The First Affiliated Hospital of Nanjing Medical University, Nanjing, PR China Guangzhou Road 300, 210029; 2 Department of Cardiology, ZhuJiang Hospital of Southern Medical University, Guangzhou, China; 3 Department of Cardiology, the Second Affiliated Hospital of Xi’an JiaoTong University, Xi’an, China; 4 Department of Cardiology, the First Hospital of Lanzhou University, Lanzhou, China

**Keywords:** Hypertrophic cardiomyopathy, Ventricular tachycardia, Catheter ablation

## Abstract

**Background:**

Ventricular tachycardia (VT) is the primary cause of sudden cardiac death in patients with hypertrophic cardiomyopathy (HCM). However, the strategy for VT treatment in HCM patients remains unclear. This study is aimed to compare the effectiveness of catheter ablation versus antiarrhythmic drug (AAD) therapy for sustained VT in patients with HCM.

**Methods:**

A total of 28 HCM patients with sustained VT at 4 different centers between December 2012 and December 2021 were enrolled. Twelve underwent catheter ablation (ablation group) and sixteen received AAD therapy (AAD group). The primary outcome was VT recurrence during follow-up.

**Results:**

Baseline characteristics were comparable between two groups. After a mean follow-up of 31.4 ± 17.5 months, the primary outcome occurred in 35.7% of the ablation group and 90.6% of the AAD group (hazard ratio [HR], 0.29 [95%CI, 0.10–0.89]; *P* = 0.021). No differences in hospital admission due to cardiovascular cause (25.0% vs. 71.0%; *P* = 0.138) and cardiovascular cause-related mortality/heart transplantation (9.1% vs. 50.6%; *P* = 0.551) were observed. However, there was a significant reduction in the composite endpoint of VT recurrence, hospital admission due to cardiovascular cause, cardiovascular cause-related mortality, or heart transplantation in ablation group as compared to that of AAD group (42.9% vs. 93.7%; HR, 0.34 [95% CI, 0.12–0.95]; *P* = 0.029).

**Conclusions:**

In HCM patients with sustained VT, catheter ablation reduced the VT recurrence, and the composite endpoint of VT recurrence, hospital admission due to cardiovascular cause, cardiovascular cause-related mortality, or heart transplantation as compared to AAD.

## Introduction

Hypertrophic cardiomyopathy (HCM) is a genetic disease of the sarcomere characterized by abnormal left ventricular (LV) hypertrophy [1]. Previous studies have reported that HCM is one of the most common heart diseases in patients with sudden cardiac death (SCD) [2]. SCD due to ventricular tachycardia (VT) is the most devastating consequence of HCM [3]. Although implantable cardioverter-defibrillators (ICD) have aborted many SCD events [4], a number of HCM patients have frequent shocks due to VT, which decrease the quality of life and increase the mortality [5, 6]. In patients with ischemic cardiomyopathy (ICM), previous studies have shown that both catheter ablation and antiarrhythmic drugs (AADs) therapy reduce appropriate ICD shocks [7, 8]. The long-term outcome post-ablation for VT in patients with non-ischemic cardiomyopathy (NICM) was significantly worse than in those with ICM because of the complexity of the underlying arrhythmogenic substrates in patients with NICM [9, 10]. A previous study has suggested different outcomes for patients with various types of NICM, HCM reported to have relatively poorer outcomes [11]. AAD is another palliative therapy for management of VT in patients with HCM, with limited efficacy and significant side effects [12]. However, little is known about the outcomes of catheter ablation versus AAD therapy for sustained VT in patients with HCM.

The objective of this study is to compare the clinical characteristics and outcomes of catheter ablation versus AAD therapy for sustained VT in patients with HCM.

## Methods

### Study population

The study population includes 28 consecutive HCM patients with sustained VT admitted to the 4 different centers between December 2012 and December 2021. Twenty-three (82.1%) patients had a history of ICD implantation for secondary prevention. The diagnosis of HCM was based on the demonstration of an unexplained left ventricle (LV) hypertrophy (LV maximum wall thickness ≥ 15 mm) in the absence of other causes of significant hypertrophy on echocardiography or magnetic resonance imaging [13]. The coronary artery disease was excluded by coronary angiography or multidetector computed tomography. The exclusion criteria included patients with prior VT ablation, patients with malignant tumors or hematological diseases with a life expectancy of less than 1 year, patients with incomplete data and patients who didn’t visit for routine follow-up.

Patients enrolled in the present study were assigned to ablation group or AAD group determined by a combination of the physician’s judgments and patients’ choices on admission. The study was carried out in accordance with the principles of the Declaration of Helsinki and was approved by the ethics committee of the participating centers. Informed consent was obtained from all patients. The study was registered in the Chinese Clinical Trial Registry (ChiCTR2300076819, 19/10/2023).

### Ablation group

The procedure was performed under general anesthesia. A quadripolar electrode catheter was placed in the right ventricular (RV) apex and the RV outflow tract for ventricular stimulation. Programmed ventricular stimulation was delivered with up to three extra stimuli to induce clinical VT if it was not present at baseline. VT mapping and catheter ablation were performed after the ventricular stimulation. Electro-anatomical mapping was performed using Carto 3 (Biosense Webster, Diamond Bar, CA, USA), or Ensite NavX (St. Jude Medical, St. Paul, MN, USA), or Rhythmia (Boston Scientific, Natick, MA, USA) system. When the VT induced was hemodynamically stable, activation and entrainment mapping were used to identify and ablate the critical pathway of the VT circuit. When the VT induced was hemodynamically unstable, the ablation was then guided by substrate mapping and/or pace mapping to define the critical region. A bipolar voltage amplitude > 1.5 mV was defined as normal myocardium and a voltage ≤ 1.5 mV as electrophysiological scar [14]. Areas of low-voltage, fractionated or late electrograms were tagged and ablated.

Endocardial mapping and ablation were initially performed. In those cases where endocardial ablation failed, epicardial ablation was performed subsequently. Epicardial access was achieved using the percutaneous subxiphoid approach described by Sosa et al. [15]. Coronary angiography was performed prior to epicardial ablation in order to avoid injuries to the coronary arteries, and high-output pacing was performed for phrenic nerve localization. Ablation was done using an irrigated-tip ablation catheter (Thermocool Smarttouch / Cool flex). Radiofrequency energy was delivered with a power of 30–40 W, temperature limit of 43 ℃, and flow of 30-30 ml/min. Following the ablation, ventricular programmed stimulation was repeated up to three extra stimuli with isoproterenol infusion in all patients. Acute success was defined as no inducibility of targeted VTs and elimination of abnormal potentials.

### AAD group

Patients in the AAD group received beta-blocker (metoprolol 47.5 mg/d or 23.75 mg/d) and/or amiodarone (200 mg/d). The dose of metoprolol would be adjusted based on the mean sinus heart rate as per the 24-hour Holter. If one patient had not received amiodarone on admission, the patient was initially given the loading dose of 600 mg/d for 10 days, followed by the maintenance dose of 200 mg/d. In addition, mexiletine was selectively given according to the patients’ specific condition.

### Endpoints and follow-up

The primary endpoint was VT recurrence. The secondary endpoints included: (1) hospital admission due to cardiovascular (CV) cause; (2) CV cause-related mortality, or heart transplantation; (3) the composite endpoint of VT recurrence, hospital admission due to CV cause, CV cause-related mortality, or heart transplantation.

VT recurrence was defined as any appropriate ICD therapy (shock or anti-tachycardia pacing) or documented sustained VT (≥ 30 s) by electrocardiogram (ECG) / 24-hour Holter monitoring. Hospital admission due to CV cause was defined as a hospital readmission for manifestations or complications of heart failure, procedure-associated complications, or arrhythmic events during follow-up. Follow-up for VT recurrence included routine office visits and device interrogations. Patients were evaluated at 3 months after discharge and then at 6- to 12-month intervals. The majority of patients (7/12, 58.3%) in the ablation group and all patients of AAD group had implanted ICD on admission. For patients without implanted ICD, 24-hour Holter was performed at 3, 6,12 months after discharge and then at 12-month intervals, or whenever arrhythmia symptoms appeared.

### Statistical analysis

Continuous variables with a normal distribution were presented as mean ± standard deviation (SD) and were compared using the two-sample t test. Continuous variables without a normal distribution were presented as median with inter-quartile range (IQR) and were compared using the Mann Whitney U test. Categorical variables were presented as frequency (percentage) and were analyzed using the Fisher’s exact test. Kaplan-Meier analysis was used to estimate the event-free survival rates, and log-rank test was used to compare event-free survival differences. Hazard ratios (HRs) and confidence intervals (CIs) were calculated with Cox proportional hazards models. A two-sided *p*-value < 0.05 was considered statistically significant.

A recent meta-analysis from six studies consisted of 68 HCM patients with VT who underwent catheter ablation, the pooled incidence of freedom from recurrent VT after the index procedure was 70.2% [16]. Considering the limited effectiveness of AADs in patients with NICM and VT, the incidence of freedom from recurrent VT treated with AAD in HCM was estimated as 20% [10]. Because it is easy to get more patients in AAD group, the ratio was set as 1:1.5. A sample size of 25 patients, grouped in 1:1.5 ratio, would provide 80% power to detect a 50% increase in the primary endpoint, with an alpha of 0.05. Allowing for a dropout rate of 10%, at least 28 patients (12 in ablation group and 16 in AAD group) should be included in the present study.

## Results

### Patient characteristics

The baseline demographic and clinical characteristics of the patients were listed in Table 1. Among 28 HCM patients with sustained VT, the mean age was 55.4 ± 11.6 years, and 26 (92.9%) patients were male. Eight patients (28.6%) had a history of syncope, three (10.7%) had a family history of HCM, and three (10.7) had a family history of SCD. The average left ventricular ejection fraction (LVEF) was 52.7 ± 12.8%, 11 (39.3%) had a LVEF < 50%, and 19 (67.9%) were classified as New York Heart Association class I to II.

**Table 1:** Baseline characteristics

	All (*n* = 28)	Ablation group (*n* = 12)	AAD group (*n* = 16)	*P* value
Age (year)	55.4 ± 11.6	52.5 ± 9.6	57.6 ± 12.7	0.261
Male (%)	26 (92.9)	12 (100)	14 (87.5)	0.492
Family history of HCM (%)	3 (10.7)	1 (8.3)	2 (12.5)	1.000
Family history of SCD (%)	3 (10.7)	2 (16.7)	1 (6.3)	0.560
History of syncope (%)	8 (28.6)	3 (25.0)	5 (31.3)	1.000
Comorbidities (%)				
Hypertension	13 (46.4)	7 (58.3)	6 (37.5)	0.445
Diabetes mellitus	3 (10.7)	1 (8.3)	2 (12.5)	1.000
Coronary heart disease	5 (17.9)	2 (16.7)	3 (18.8)	1.000
Atrial fibrillation	5 (17.9)	2 (16.7)	3 (18.8)	1.000
NYHA functional class (%)				0.687
I / II	19 (67.9)	9 (75.0)	10 (62.5)	
III / IV	9 (32.1)	3 (25.0)	6 (37.5)	
Transthoracic echocardiography				
LVEDD (mm)	53.4 ± 8.8	51.8 ± 7.7	54.6 ± 9.6	0.417
LVESD (mm)	37.7 ± 11.3	34.0 ± 11.1	40.3 ± 11.1	0.161
IVS (mm)	15.5 ± 4.9	14.8 ± 5.1	16.0 ± 1.3	0.537
LVPW (mm)	10.8 ± 1.5	11.2 ± 1.8	10.5 ± 1.3	0.247
LVEF (%)	52.7 ± 12.8	54.5 ± 10.4	51.5 ± 14.6	0.548
LVEF < 50%	11 (39.3)	4 (33.3)	7 (43.8)	0.705
LVOT obstruction (%)	7 (25.0)	5 (41.7)	2 (12.5)	0.103
Apical HCM (%)	3 (10.7)	0	3 (18.8)	0.238
VT storm (%)	6 (21.4)	4 (33.9)	2 (12.5)	0.354
History of ventricular septal alcohol ablation (%)	4 (14.3)	3 (25.0)	1 (6.3)	0.285
History of ventricular septum surgical excision (%)	1 (3.6)	1 (8.3)	0	0.429
Number of previous VT episodes (n)	3 (2, 3)	3 (2, 4.5)	3 (2, 3)	0.631
Polymorphic VT (%)	4 (14.3)	2 (16.7)	2 (12.5)	1.000
VT cycle length (ms)	309 ± 31	312 ± 30	307 ± 33	0.710
The number of previous used AADs (n)	2.0 ± 0.6	2.1 ± 0.7	1.9 ± 0.6	0.540
Medications at the entry of study (%)				
Beta-blocker (%)	28 (100)	12 (100)	16 (100)	-
Amiodarone (%)	17 (60.7)	7 (58.3)	10 (62.5)	1.000
Other AAD (%)	5 (17.9)	2 (16.7)	3 (18.8)	1.000

*AAD *Antiarrhythmic drug, *HCM *Hypertrophic cardiomyopathy, *SCD *Sudden cardiac death, *NYHA *New York Heart Association, *LVEDD *Left ventricular end-diastolic diameter, *LVESD *Left ventricular end-systolic diameter, *IVS *Interventricular septum, *LVPW *Left ventricular posterior wall, *LVEF *Left ventricular ejection fraction, *LVOT *Left ventricular outflow tract, *LV *Left ventricle, *VT *Ventricular tachycardia

Six (21.4%) patients had a history of VT storm. Five (17.9%) patients had a history of atrial fibrillation. Seven (25.0%) had an obstructive left ventricular outflow tract (LVOT) with a pressure gradient of ≥ 30 mmHg. Four had a history of ventricular septal alcohol ablation and one had a history of ventricular septum surgical excision. On admission, all patients had a history of using beta-blockers, 17 (60.7%) had a history of using amiodarone, and 5 (17.9%) had a history of using mexiletine. Among 28 HCM patients with sustained VT, 12 underwent catheter ablation (ablation group) and 16 received AAD therapy (AAD group). All baseline characteristics were comparable between two groups (Table 1).

### Mapping and ablation of sustained ventricular tachycardias

Table 2 summarizes the electrophysiological and procedural characteristics of the patients in the ablation group. With programmed ventricular stimulation, sustained VT was induced in 8 patients. Among 8 VTs, 2 VTs were then transformed into ventricular fibrillation (VF) with hemodynamically unstable state. Among the stable VTs induced, the mean cycle length was 332 ms (limits, 260 to 480 ms). LV apical aneurysm was observed in 4 (33.3%) patients.

**Table 2:** Procedural characteristics and outcomes

No.	Procedure time (min)	Ablation time (min)	VT cycle length (ms)	VT Induction (%)	LV apical aneurysm	Mapping location	Bipolar low voltage (Substrate mapping)	Activation mapping	Entrainment mapping	Pace mapping
1	255	32	300	Yes	No	Endo	Endo: LV apex, base and free wall	–	–	+
2	170	30	260,320	No	Yes	Endo + Epi	Endo: LV apex	–	–	+
3	166	25	350	Yes	No	Endo + Epi	Endo + Epi: LV apex	+	–	+
4	392	35	250	Yes (VF)	Yes	Endo + Epi	Endo + Epi: LV apex	–	–	+
5	350	22	300	Yes	Yes	Epi	Epi: LV anterior wall	+	–	+
6	220	30	330	Yes	Yes	Endo	Endo: LV apical aneurysm	+	–	+
7	240	25	315	Yes	No	Endo + Epi	Epi: LV anterior wall	+	+	–
8	250	36	350	No	No	Endo + Epi	Endo + Epi: LV apex near free wall	–	–	–
9	330	60	260,280	Yes (VF)	No	Endo + Epi	Endo: LV anterior, RV inferior wall Epi: LV apex, lateral wall, RV base and inferior wall	–	–	–
10	280	40	310	No	No	Endo + Epi	Epi: roof of LV, anterior interventricular septum	–	–	–
11	240	28	260	Yes	No	Epi	Epi: LV anterior and inferior wall	+	–	–
12	360	33	320	No	No	Endo + Epi	Endo: junction of LV roof and anterior wall Epi: LV roof and lateral wall	–	–	–

*VT *Ventricular tachycardia, *LV *Left ventricle, *RV *Right ventricle, *Endo *Endocardial, *Epi *Epicardial

**Fig. 1:**
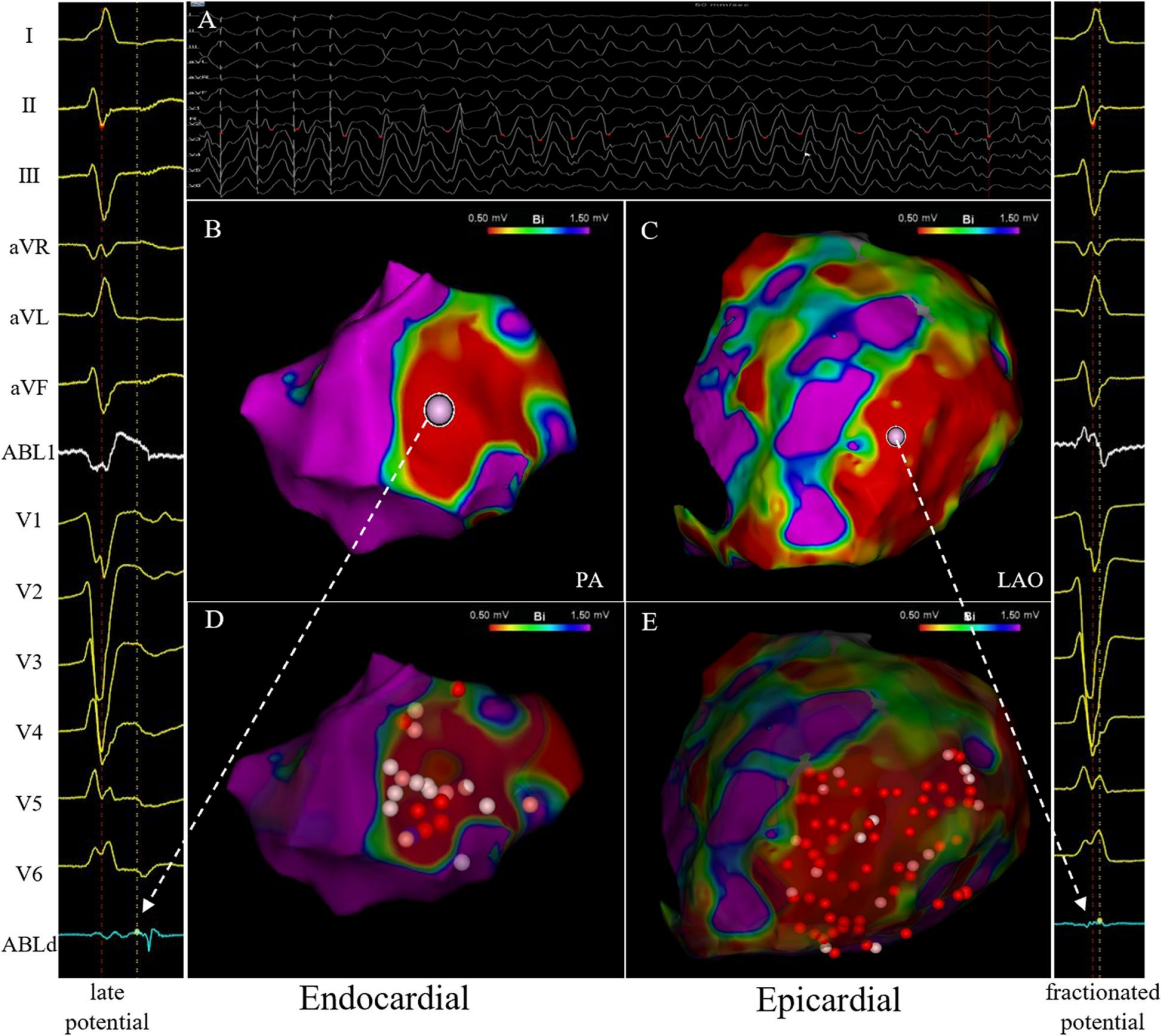
Twelve-lead electrocardiogram, endocardial and epicardial bipolar voltage maps and radiofrequency ablation. **A** Ventricular stimulation induced ventricular tachycardia (VT), which was then transformed into ventricular fibrillation (VF), along with hemodynamically unstable state. Electrical defibrillation was performed immediately to convert to sinus rhythm, followed by voltage mapping. **B** Endocardial low voltage of left ventricle (LV) free wall near LV apex in the posterior anterior (PA) view. The arrow in the panel B shows late potential. **C** Epicardial low voltage of LV free wall near LV apex in the left anterior oblique (LAO) view. The arrow in the panel **C** shows fractionated potential. **D** and **E** show the example of radiofrequency ablation in the left ventricular endocardium (**D**) and epicardium (**E**)

Both endocardial and epicardial mapping were performed in 8 patients, and only endocardial mapping was performed in 2 patients. Only epicardial mapping in 2 patients was performed because the physician determined the epicardial origin on the basis of the ECG of clinical VT. All patients were presented with low voltage in substrate mapping by endocardial and/or epicardial approaches. The electro-anatomical bipolar voltage maps identified endocardial scar in 8 patients, and epicardial scar in 9 patients only. The most common location of scar was the LV apex in 7 (58.3%) patients, followed by LV anterior wall in 5 (41.7%) patients. Areas of abnormal potentials including late potentials (LPs) and fractionated potentials were also eliminated. Figure 1 shows an example of endocardial and epicardial low voltage maps in the LV free wall near LV apex. The late potential and fractionated potential were presented in the areas of scar. After ablation of substrate and abnormal potentials, VT was non-inducible. Except substrate mapping, pacing mapping was also performed in 6 patients without induced stable VT. Activation mapping was performed in 5 patients and entrainment mapping was performed in one patient.

VTs of 4 patients were terminated during ablation (2 in endocardial and 2 in epicardial), and one VT was terminated in the epicardial surface by mechanical stimulation. Combined endocardial and epicardial ablation was performed in 11 (91.7%) patients, while only one (8.3%) underwent endocardial-only ablation. The most common effective ablation site was the LV apex or near LV apex in 8 (66.7%) patients, followed by LV anterior wall in 5 (41.6%) patients. At the end of the procedure, non-inducibility of any VT was achieved in 10 (83.3%) patients, while non-clinical VT was induced in one (8.3%), and VT inducibility was not tested in one (8.3%). No major complications were observed in the perioperative period in all patients except one transient third-degree atrioventricular block.

### Antiarrhythmic drug usage

On discharge, all patients in the AAD group received beta-blocker (metoprolol 47.5 mg/d or 23.75 mg/d) and amiodarone (loading dose of 600 mg/d for 10 days followed by a maintenance dose of 200 mg/d).

On the last follow-up visit, all patients except one who underwent heart transplantation in the AAD group received beta-blockers, 10 (62.5%) patients received amiodarone, and one patient received mexiletine. In the ablation group, 10 (83.3%) patients received beta-blockers, 3 (25.0%) received amiodarone, one received mexiletine, and one did not use any AAD due to heart transplantation. The amiodarone usage in the ablation group was lower than that in the AAD group (25.0% vs. 62.5%, *P* = 0.067) on the last follow-up (Table 3).

**Table 3:** Long-term outcomes

	All (*n* = 28)	Ablation group (*n* = 12)	AAD group (*n* = 16)	HR (95%CI)	*P* value
Follow-up (months)	31.4 ± 17.5	31.9 ± 20.0	31.1 ± 16.1		0.908
VT recurrence (%)	18 (64.3)	4 (33.3)	14 (87.5)	0.29 (0.10–0.89)	0.021
ICD shock	14 (50.0)	2 (16.7)	12 (75.0)		0.006
Anti-tachycardia pacing	16 (57.1)	3 (25.0)	13 (81.3)		0.006
VT below ICD detection	2 (7.1)	1 (8.3)	1 (6.3)		1.000
CV hospitalization (%)	12 (42.9)	3 (25.0)	9 (56.3)	0.38 (0.10–1.43)	0.138
CV death / heart transplantation (%)	4 (14.3)	1 (8.3)	3 (18.8)	0.50 (0.05–4.99)	0.551
Composite endpoint (%)	20 (71.4)	5 (41.7)	15 (93.8)	0.34 (0.12–0.95)	0.029
Amiodarone use at last follow-up (%)	13 (46.4)	3 (25.0)	10 (62.5)		0.067
Beta-blocker use at last follow-up (%)	25 (89.3)	10 (83.3)	15 (93.8)		0.560
Mexiletine use at last follow-up (%)	2 (7.1)	1 (8.3)	1 (6.3)		1.000

*AAD *Antiarrhythmic drug, *VT *Ventricular tachycardia, *ICD *Implantable cardioverter-defibrillator, *CV *Cardiovascular, *HR *Hazard ratio

**Fig. 2:**
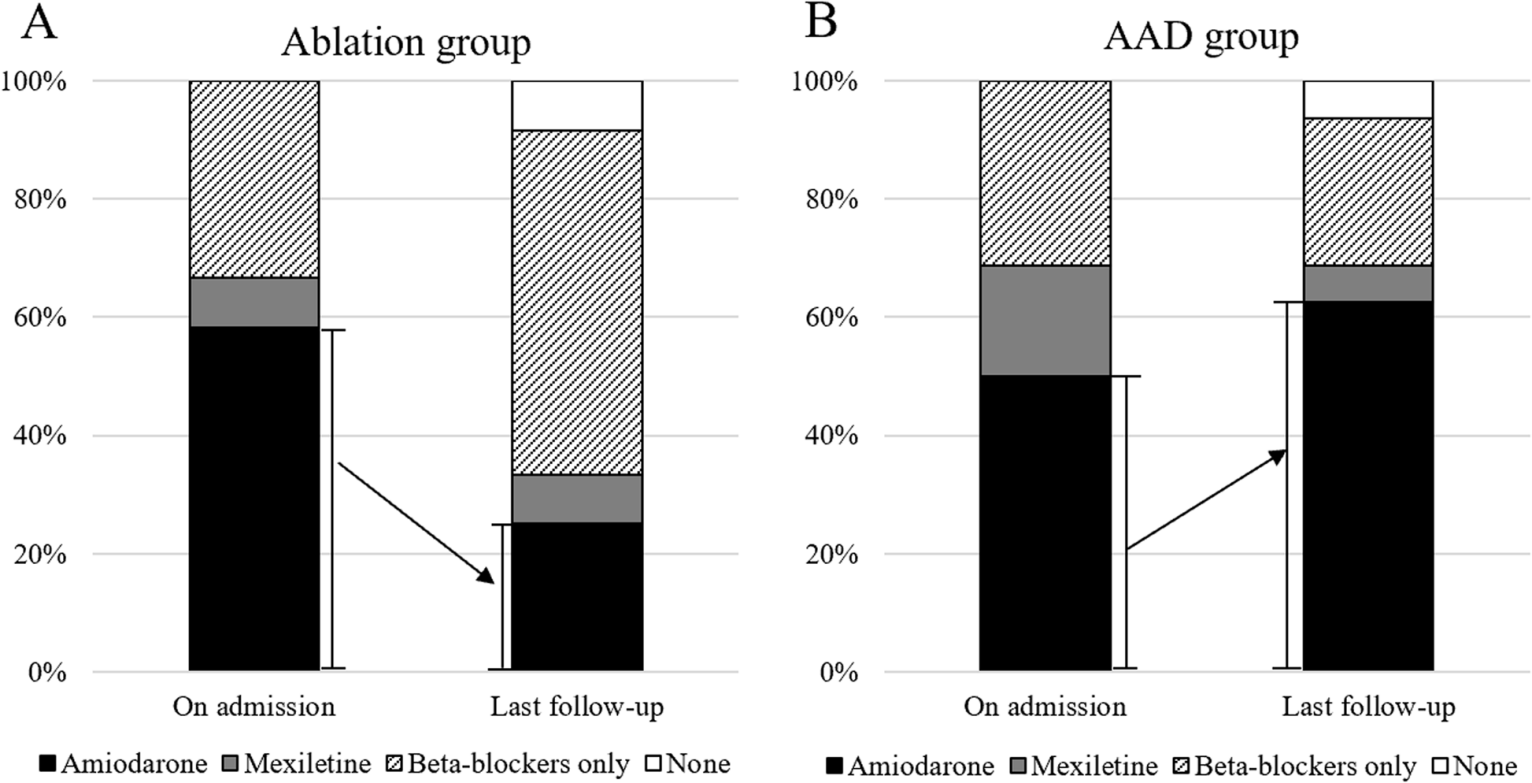
Antiarrhythmic drug usage on admission and on the last follow-up in the ablation group (**A**) and the AAD group (**B**). The left bar chart (**A**) shows a significant reduction in amiodarone usage in the ablation group with the majority of patients taking beta-blockers only on the last follow-up. The right bar chart (**B**) shows an increase in amiodarone usage in the AAD group with the majority of patients taking amiodarone on the last follow-up. AAD, antiarrhythmic drug

Compared with the AAD usage on admission, there was a reduction in amiodarone usage in the ablation group but an increase in amiodarone usage in the AAD group on the last follow-up (Fig. 2).

### Long-term outcome

**Fig. 3:**
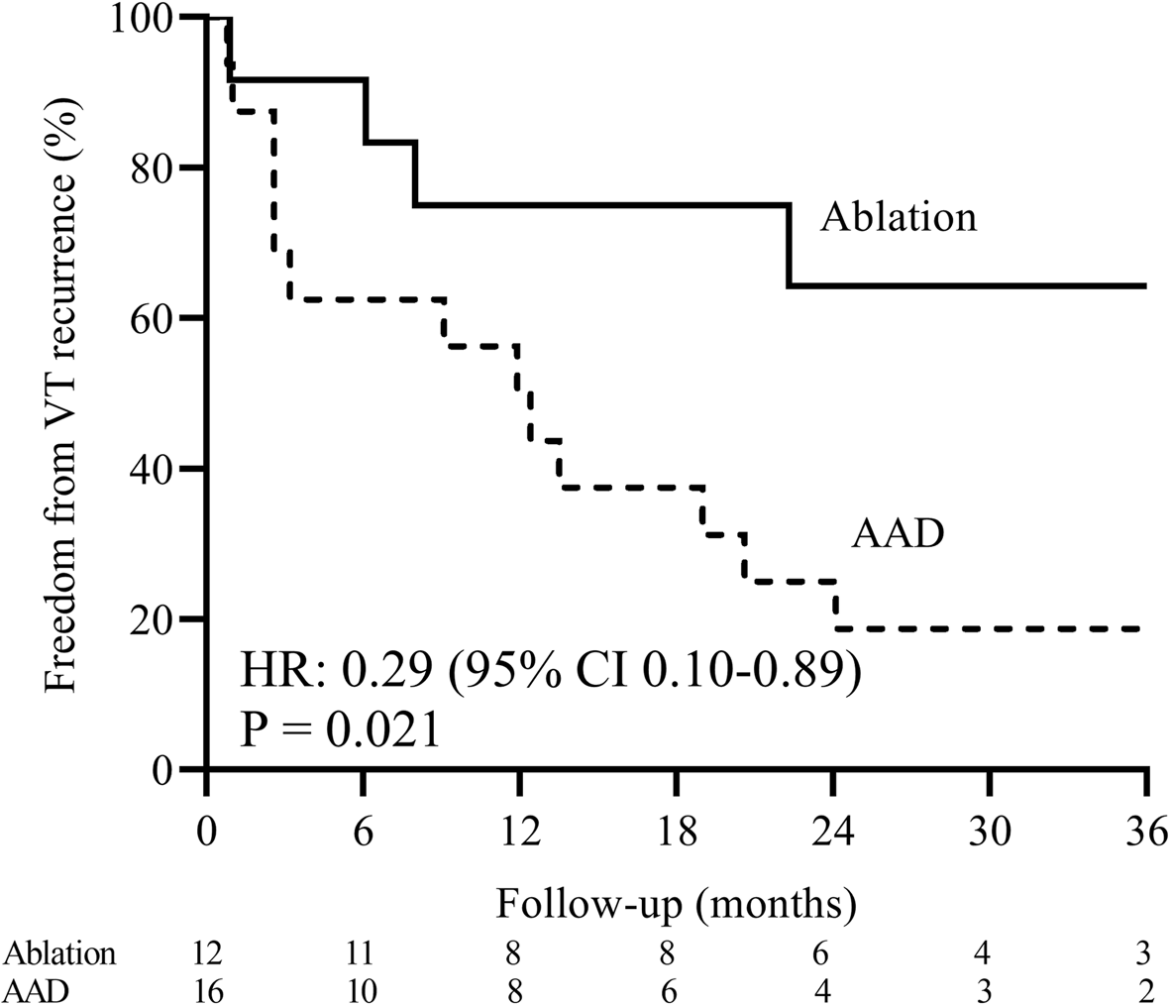
The primary outcome of the two groups. Kaplan-Meier survival curve shows freedom from the VT recurrence during follow-up. AAD, antiarrhythmic drug; VT, ventricular tachycardia

**Fig. 4:**
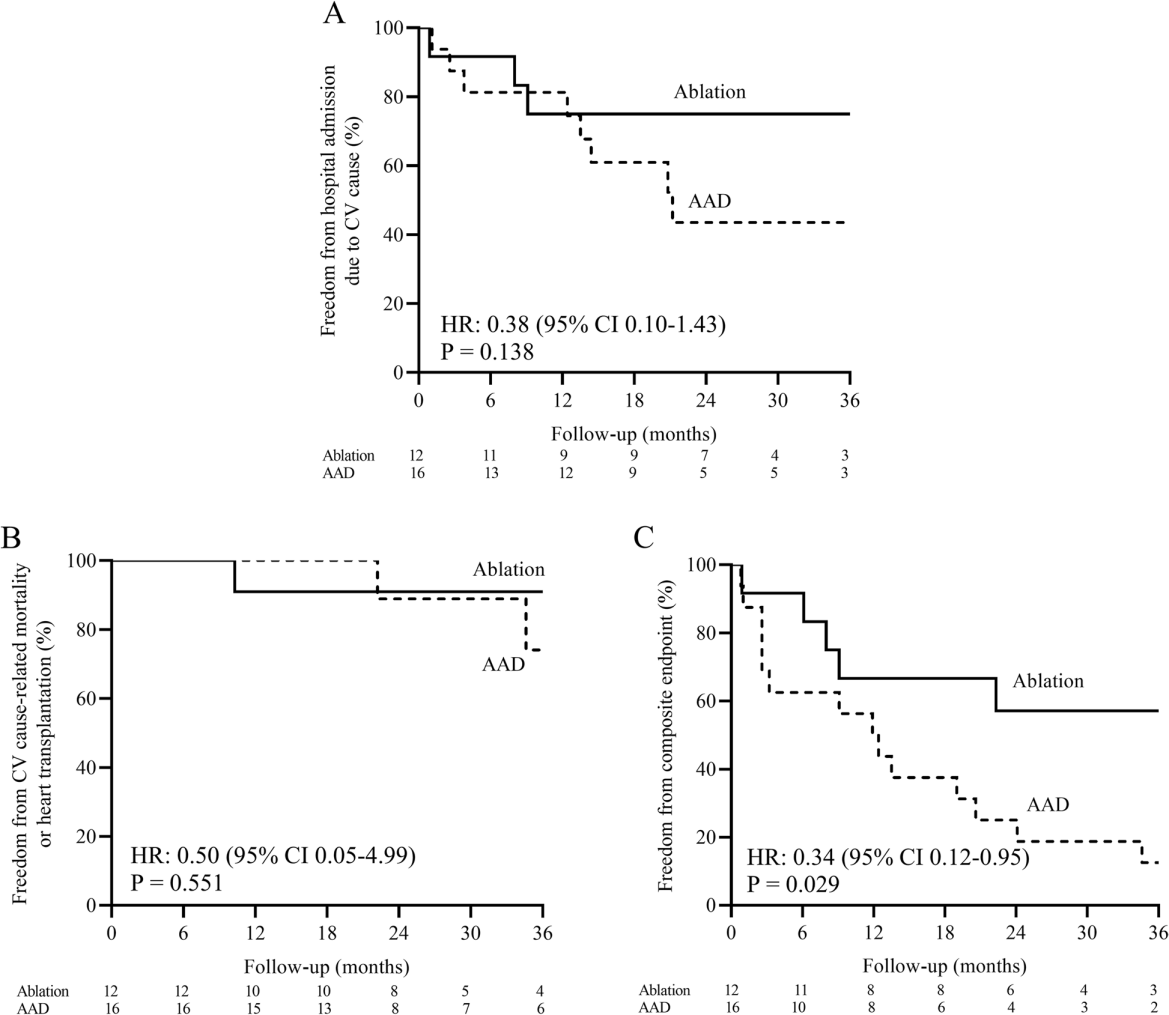
The secondary outcomes of the two groups. Kaplan-Meier survival curves show freedom from the hospital admission due to CV cause (**A**), CV cause-related mortality / heart transplantation (**B**), and the composite endpoint of VT recurrence, hospital admission due to CV cause and CV cause-related mortality / heart transplantation (**C**) during follow-up. AAD, antiarrhythmic drug; CV, cardiovascular

After a mean follow-up of 31.4 ± 17.5 months, 18 (64.3%) patients developed VT recurrence, 20 (71.4%) patients reached the composite endpoint. Hospital admission due to CV cause and CV cause-related mortality/ heart transplantation occurred in 12 (42.9%) and 4 (14.3%) patients, respectively. Kaplan-Meier survival curve shows that the VT recurrence occurred in 35.7% of the ablation group and 90.6% in the AAD group (hazard ratio [HR], 0.29 [95%CI, 0.10–0.89]; *P* = 0.021; Fig. 3; Table 3). There was a statistically significant reduction in appropriate ICD shock (16.7% versus 75.0%; *P* = 0.006) in the ablation group compared to that of the AAD group. By Kaplan-Meier estimate, hospital admission due to CV cause occurred in 25.0% of the ablation group and 71.0% of the AAD group [HR, 0.38 (95%CI, 0.10–1.43); *P* = 0.138; Fig. 4A]. CV cause-related mortality / heart transplantation occurred in 9.1% of the ablation group and 50.6% of the AAD group [HR, 0.50 (95%CI, 0.05–4.99); *P* = 0.551; Fig. 4B]. However, there was a significant reduction in the composite endpoint of VT recurrence, hospital admission due to CV cause, CV cause-related mortality, or heart transplantation in ablation group as compared to that of AAD group. (42.9% vs. 93.7%; HR, 0.34 [95% CI, 0.12–0.95]; *P* = 0.029; Fig. 4C; Table 3).

## Discussion

### Major findings

This study has shown that, in patients with HCM and symptomatic VT, catheter ablation was associated with a significantly lower rate of VT recurrence, and the composite endpoint of VT recurrence, hospital admission due to CV cause, CV cause-related mortality, or heart transplantation. However, catheter ablation did not reduce the rate of hospital admission due to CV cause, CV cause-related mortality, or heart transplantation compared to AAD.

Current guidelines recommend that, in patients with HCM and symptomatic drug refractory sustained VT, catheter ablation can be useful for reducing VT burden and ICD shocks (IIa) [13, 17]. The ablation for VT in patients with HCM is challenging. The ablation experience for VT in patients with HCM has been limited to case reports and small case series of selected patients till date. Santangeli et al. [18] reported that elimination of VTs reached 73% in 22 patients (18 with ICD) during a follow-up of 20 ± 9 months. Dukkipati et al. [19] reported that in HCM patients with VT, the freedom from recurrent ICD shocks was 78% (7/9) during a follow-up of 37 ± 17 months post-ablation. A recent meta-analysis from six studies consisted of a total of 68 drug-refractory HCM patients who underwent VT radiofrequency catheter ablation [16]. During a follow‐up of 18.3 ± 11.7 months, the pooled incidence of freedom from recurrent VT after index procedure was 70.2% (95% CI: 51.9 − 86.2%) [16]. The success rates reported by these studies were similar to that (64.3%) during a mean follow-up of 31.4 ± 17.5 months in our study. However, Naeemah et al. [20]. reported a lower (45%) success rate in 11 HCM patients with VT post-ablation with a median follow-up of 13 months. All these (11) patients were patients with dilated-phase HCM (D-HCM), which may indicate more complex arrhythmogenic substrates in patients with D-HCM. The VT recurrence in patients with HCM may be driven by intramural or epicardial scar, often necessitating combined endocardial and epicardial ablation procedures to eliminate the VT [17, 21, 22]. The epicardial mapping and ablation for VT is needed in most patients with HCM [18, 19, 23]. In the present study, combined endocardial and epicardial ablation was performed in 11 (91.7%) patients, while only one patient (8.3%) underwent endocardial‐only ablation. Epicardial scar was present in 75% of the patients, which was similar to 80% reported by Dukkipati et al. [19]. Dukkipati et al. [19]. reported that the most common location of scar in this study was LV apex (70%), which was also consistent with our study (66.7%). However, other studies reported that the location of VT scar was mostly distributed at the basal septum in HCM patients in dilated-phase [18, 20]. This may suggest that arrhythmogenic scar locations differ as HCM progresses to D-HCM, which deserves to be further studied.

Previous studies have indicated that, in patients with structural heart diseases and sustained VT, both AAD therapy and catheter ablation can reduce VT burden and ICD shocks [8, 24–26]. Since few studies have been conducted on AAD therapies to prevent ICD shocks specifically in HCM patients, the result of HCM patients is extrapolated from studies that enrolled different disease substrates. The OPTIC trial suggested that ICD shocks occurred in 38.5% of the patients assigned to beta-blocker alone and 10.3% of the patients assigned to amiodarone plus beta-blocker [8]. Amiodarone in addition to beta-blocker was more effective but at the expense of increased side effects. Only 40 (9.7%) of 412 patients had a history of NICM in this trial. Another ALPHEE study showed that in prevention of ICD shocks, amiodarone had a statistically significant, 16% absolute risk and 26% relative risk reduction as compared to placebo [24]. In the ALPHEE study, only 13 patients receiving amiodarone had a history of NICM. The present study enrolled 16 HCM patients receiving AAD therapy, and suggested that only 9.4% of patients had no recorded VT episodes. The limited efficacy of AAD in the present study may be attributed to the poor drug response in HCM patients and a high drug discontinuation rate due to the cardiac and extra-cardiac side effects of amiodarone.

Recently, the SURVIVE-VT trial [25] and the PAUSE-SCD trial [26] demonstrated that catheter ablation for VT improved the outcomes compared to AAD therapy in patients with ICM / NICM, which was consistent with our study of patients with HCM. Although catheter ablation reduced VT recurrence, no significant difference were observed in hospital admission due to CV cause and CV cause-related mortality / heart transplantation, which was also consistent with the SURVIVE-VT trial [25] and the PAUSE-SCD trial [26].

Although current guidelines [13, 17] recommend that patients with HCM should be considered for secondary prevention ICD implantation if they have a previously documented sustained VT, many patients did not receive ICD implantation in the real world [27]. In the present study, five (17.9%) patients did not have a history of ICD implantation. The most common reason for not receiving ICD implantation was that three patients could not afford the device cost. The other reasons were that one patient unwilling to bear the risks associated with implantation and one patient not believing in the benefit of ICD.

### Limitations

The limitations of this study should be acknowledged. First of all, this was a retrospective study that included only patients hospitalized in selectively tertiary centers. Second, selection of catheter ablation or AAD was left to the treating physicians and patients, but not randomized. However, the baseline characteristics were comparable between two groups. Third, not all the patients of ablation group received ICD implantation so that partial VT episodes were not recorded. The 24-hour Holter monitoring and ECG monitoring were performed to reduce the potential impact.

## Conclusion

In HCM patients with symptomatic sustained VT, catheter ablation reduced the VT recurrence, and the composite endpoint of VT recurrence, hospital admission due to CV cause, CV cause-related mortality, or heart transplantation compared to AAD.

## Data Availability

The data used and analyzed to support the findings of this study are available from the corresponding author on reasonable request.

## Abbreviations

AAD: Antiarrhythmic drug
CV: Cardiovascular
HCM: Hypertrophic cardiomyopathy
ICD: Implantable cardioverter-defibrillator
ICM: Ischemic cardiomyopathy
LV: Left ventricle
NICM: Non-ischemic cardiomyopathy
RV: Right ventricular
SCD: Sudden cardiac death
VT: Ventricular tachycardia
